# Effects of ovariectomy‐induced osteoporosis and two photobiomodulation protocols on the repair of apical periodontitis in Wistar rats: A microCT study

**DOI:** 10.1111/iej.14234

**Published:** 2025-04-04

**Authors:** Carolina Horn Troian‐Michel, Bruna Barcelos Só, Lauren Frenzel Schuch, Grazielle Oliveira Stelter, Tuany Rafaeli Schmidt, Joana Letícia Schorr, Daniela Campagnol, Tuane Nerissa Alves Garcez, Gabriel Barcelos Só, Thalita Ayres Arrué, Theodoro Weissheimer, Manoela Domingues Martins, Marcus Vinicius Reis Só

**Affiliations:** ^1^ Department of Endodontics School of Dentistry, Federal University of Rio Grande Do Sul Porto Alegre Brazil; ^2^ Department of Oral Pathology School of Dentistry, Federal University of Rio Grande Do Sul Porto Alegre Brazil; ^3^ Department of Pathology and Oral Medicine Diagnosis School of Dentistry, Universidad de la República Montevideo Uruguay; ^4^ School of Veterinary Medicine, University of Vila Velha Vila Velha Brazil; ^5^ Experiental Research Center, Hospital de Clínicas de Porto Alegre Porto Alegre Brazil

**Keywords:** animal model, apical periodontitis, endodontic treatment, oestrogenosteoporosisPhotobiomodulation therapy

## Abstract

**Aim:**

Osteoporosis is a disease that is related to a higher prevalence and greater volumes of apical periodontitis. The purpose of this study was to evaluate the effect of ovariectomy‐induced osteoporosis and high‐ or low‐level laser photobiomodulation (PBM) on the repair of apical periodontitis (AP) in adult female Wistar rats.

**Methodology:**

Sixty female Wistar rats (*n* = 120) were randomly allocated into four control (*n* = 6 teeth) and 8 experimental (*n* = 12 teeth) groups, according to procedure and time of evaluation: healthy control (GCS14/GCS28), osteoporotic control (GCO14/GCO28), sham (SHAM14/SHAM28), osteoporotic (OVX14/OVX28), low‐level laser (OVX‐LLL14/OVX‐LLL28) and high‐level laser (OVX‐GEM14/OVX‐GEM28). All groups were submitted to bilateral ovariectomy, except for the healthy control and sham. Eight weeks later, AP was induced bilaterally in mandibular first molars' mesial roots in experimental groups, and after 21 days, their endodontic treatment (RCT) was conducted. For 14 or 28 days, PBM was applied three times/week, as follows: sham application (SHAM and OVX groups); low‐level laser (OVX/LLL groups); defocused high‐power laser (OVX/GEM groups). After the respective experimental periods, the animals were euthanized. Tibias and hemi‐mandibula were obtained and submitted to computed microtomography. Bone volume fraction (BV/TV), trabeculae number (Tb.N), spacing (Tb.Sp) and thickness (Tb.Th) were obtained for tibias, while RCT apical limit, periodontal ligament and AP volumes (APvol) were recorded for hemi‐mandibula. Data were subjected to statistical analysis through generalized estimating equations (GEE), ANOVA and chi‐square tests (5% significance level).

**Results:**

Ovariectomy tibias showed lower BV/TV and Tb.N (*p* < .0001) and higher Tb.Th (*p* = .020) and Tb.Sp (*p* < .0001) when compared to sham groups. For hemi‐mandibula, lower APvol values were found in SHAM14 when compared to OVX/LLL14 and OVX/GEM14 groups (*p* < .05), which were not different from each other. Over the 28‐day period, the OVX/LLL28 group showed higher AP volumes when compared to OVX28 and OVX/GEM28 (*p* < .05). Analysing variables effects on APvol, time, PBM and RCT apical limit showed no effect (*p* > .05), while ovariectomy showed a significant increase (*p* = .004).

**Conclusions:**

Ovariectomy‐induced osteoporosis in Wistar rats led to a reduction in AP repair, and PBM was unable to counteract this effect.

## INTRODUCTION

Osteoporosis is a major public health problem. It is the most common bone disease in humans, characterized by reduced mass, tissue deterioration and disruption of bone architecture, compromising strength and increasing the risk of fractures (Cosman et al., 2014). It is mainly caused by a decrease in oestrogen levels, which is a characteristic of post‐menopausal women, and has a major impact on oral health (Scardina & Messina, 2012). In sites with an inflammatory process, it might exacerbate bone resorption and influence bone remodelling, since the cytokines involved in inflammation‐induced remodelling are similar to those that seem to play a critical role in postmenopausal osteoporosis (Lerner, 2006). Several studies have shown that osteoporosis is considered a risk factor for the development and progression of periodontal disease due to increased propensity for bone loss (Geurs et al., 2003; Guiglia et al., 2013; Kim et al., 2015; Richa et al., 2017; Tezal et al., 2000). Furthermore, it is related to delay in socket repair following tooth extraction (Arioka et al., 2019; Chen et al., 2018; Luvizuto et al., 2011; Luvizuto, Dias, et al., 2010; Luvizuto, Queiroz, et al., 2010; Pereira et al., 2007; Ramalho‐Ferreira et al., 2017).

Apical periodontitis (AP) is an inflammatory disease with a prevalence of 52% around the world (Tibúrcio‐Machado et al., 2021). It is characterized by an immunopathological response from the body in reaction to the invasion of microorganisms within the root canal system, leading to bone resorption (Kakehashi et al., 1965). This process is mediated by several proinflammatory cytokines secreted by cells recruited in response to microbial infection from the root canal system; although the exact mechanism of AP is complex and not fully elucidated, it is known that tumour necrosis factor alpha (TNF‐α), interleukin 1 beta (IL‐1β) and interleukin‐6 (IL‐6) contribute to the differentiation of osteoclasts and periapical bone resorption (Kawashima & Stashenko, 1999; Qian et al., 2016; Wei et al., 2013). The pathogenesis of periapical diseases may also be influenced by some systemic factors, among them hormones (Qian et al., 2016; Xiong et al., 2007).

The clinical association between osteoporosis and AP has been the subject of some studies (Cadoni et al., 2022; Freitas et al., 2024; Katz & Rotstein, 2021; López‐López et al., 2015). They found a positive correlation between them, with a tendency towards a higher prevalence of AP in osteoporotic patients. Furthermore, there are several experimental studies in animal models that induced, through ovariectomy (OVX), a decrease in oestrogen levels and consequent osteoporosis. The results are consistent in showing that AP in ovariectomized rats is characterized by greater periapical bone resorption when compared to lesions in healthy animals (Brasil et al., 2017; Gilles et al., 1997; Gomes‐Filho et al., 2015; Guan et al., 2020; Liu et al., 2010; Qian et al., 2016, 2020; Romualdo et al., 2018; Rossetti et al., 2022; Silva et al., 2020; Wayama et al., 2015; Xiong et al., 2007; Zhang et al., 2011). The serum levels of proinflammatory cytokines were also significantly higher in ovariectomized rats, which demonstrates the role of oestrogen in protecting against the effects of inflammation (Guan et al., 2020; Qian et al., 2016, 2020; Romualdo et al., 2018; Zhang et al., 2011).

Photobiomodulation (PBM) is a therapy that uses lasers and/or light‐emitting diodes (LED) in a non‐thermal process that involves the absorption of light photons by chromophores, triggering different responses depending on the protocol used and type of cell (Anders et al., 2015). There is an increase in energy production (ATP), coupled with a reduction in reactive oxygen species (ROS) in cells and tissues affected by oxidative stress, that might result in the modulation of inflammation, a decrease in pain, acceleration of cellular proliferation, wound healing and tissue regeneration (Hamblin, 2017). In the last decades, PBM has been used for several conditions and extensively studied. It has been shown that it increases osteoblast proliferation (Dompe et al., 2020; Miranda et al., 2020; Niimi et al., 2020; Oliveira et al., 2016; Zaccara et al., 2020), raises calcium transport during bone formation and angiogenesis (Son et al., 2017), and improves bone healing after oral surgery (Brignardello‐Petersen et al., 2012; Kulkarni et al., 2019; Lemes et al., 2019; Metin et al., 2018; Mozzati et al., 2012; Noba et al., 2018; Romão et al., 2015; Scarano et al., 2021; Zaky et al., 2016). In the presence of osteoporosis, PBM was effective in improving bone repair (Aras et al., 2015; Asgari et al., 2020; Bossini et al., 2011; Pinheiro et al., 2018; Scalize et al., 2015). In healthy patients, it accelerated periapical repair of endodontically treated teeth (Das et al., 2023; Shah et al., 2021). On the other hand, no previous study evaluated the effect of PBM on apical periodontitis healing in osteoporotic conditions. Considering the facts that most women remain untreated for osteoporosis and that females' systemic response to AP is significantly improved, it remains necessary for new therapeutic approaches to be developed to solve this condition. Therefore, the purpose of the present study is to evaluate the effect of ovariectomy‐induced osteoporosis and high‐ or low‐level laser photobiomodulation (PBM) on the repair of apical periodontitis (AP) in adult female Wistar rats.

## MATERIALS AND METHODS

The manuscript of this animal study has been written according to Preferred Reporting Items for Animal studies in Endodontology (PRIASE) 2021 guidelines (Nagendrababu et al., 2021). The Ethics Committee of Animal Use from Hospital de Clínicas de Porto Alegre (CEUA 2021‐0252) approved this study. It was conducted in the Center of Experimental Research from Hospital de Clínicas de Porto Alegre and in the Conservative Dentistry Department from the School of Dentistry of the Federal University of Rio Grande do Sul. The present study respected the national laws on animal use, and an environmental enrichment program was adopted to promote animal welfare (CONCEA RN 30/2016). Animals' overall health was monitored throughout the experiment by two veterinarians experienced in conducting animal model studies.

### Sample

A total of 60 female rats (*Rattus novergicus albinus, Wistar*) at 10 weeks of age, weighing approximately 250.87 g (±17.16), were used for this study. They were submitted to a 15‐day period of acclimatization. The animals were allocated in accommodation specific to the species, with controlled temperature (22 ± 2°C, 20–24°C), with a 12‐hour light/dark cycle, at relative humidity of 40–60%. Three animals were allocated in each cage. Animals received standard diet and water *ad libitum* before the experiments. Sample size calculation was based on comparisons between means, assuming a minimum difference of 15 units with an overall estimated standard deviation of 10 (effect size = 1.5) of alveolar bone repair from (Çırak et al., 2018) using WinPepi (WINPEPI freeware computer programs for epidemiologists), with power established at 80% and significance level at 5%. The result was 7 observations per group. Considering about 10% of sample loss, a minimum of 8 samples for each experimental group was established.

### Experimental groups

In each cage, the three animals were allocated to the same group; the cages were drawn with paper to define the group they belonged to. Animals in cages were, in this way, randomly allocated to one of the twelve following groups:

GCS14 (*n* = 6 teeth)—healthy control, sham surgery, no additional procedure, euthanasia 13 weeks after operation.

GCS28 (*n* = 6 teeth)—healthy control, sham surgery, no additional procedure, euthanasia 15 weeks after operation.

GCO14 (*n* = 6 teeth)—osteoporotic control, OVX surgery, no additional procedure, euthanasia 13 weeks after operation.

GCO28 (*n* = 6 teeth)—osteoporotic control, OVX surgery, no additional procedure, euthanasia 15 weeks after operation.

SHAM14 (*n* = 12 teeth)—healthy group, sham surgery, apical periodontitis induction, endodontic treatment, no additional procedure, euthanasia 14 days after endodontic treatment.

SHAM28 (*n* = 12 teeth)—healthy group, sham surgery, apical periodontitis induction, endodontic treatment, no additional procedure, euthanasia 28 days after endodontic treatment.

OVX14 (*n* = 12 teeth)—osteoporotic group, OVX surgery, apical periodontitis induction, endodontic treatment, no additional procedure, euthanasia 14 days after endodontic treatment.

OVX28 (*n* = 12 teeth)—osteoporotic group, OVX surgery, apical periodontitis induction, endodontic treatment, no additional procedure, euthanasia 28 days after endodontic treatment.

OVX/LLL14 (*n* = 12 teeth)—osteoporotic group, OVX surgery, apical periodontitis induction, endodontic treatment, low level laser therapy, euthanasia 14 days after endodontic treatment.

OVX/LLL28 (*n* = 12 teeth)—osteoporotic group, OVX surgery, apical periodontitis induction, endodontic treatment, low level laser therapy, euthanasia 28 days after endodontic treatment.

OVX/GEM14 (*n* = 12 teeth)—osteoporotic group, OVX surgery, apical periodontitis induction, endodontic treatment, defocused high‐power laser 1 W, euthanasia 14 days after endodontic treatment.

OVX/GEM28 (n = 12 teeth)—osteoporotic group, OVX surgery, apical periodontitis induction, endodontic treatment, defocused high‐power laser 1 W, euthanasia 28 days after endodontic treatment.

One week prior to the ovariectomy (OVX) surgeries, OVX animals started to receive a low calcium diet (Domeneghetti e Corrêa Ltda, Jaú) with 0.2% Ca and 0.53% P, according to previously described protocols recommended for osteoporosis induction (Prado et al., 2012; Ramalho‐Ferreira et al., 2017; Teófilo et al., 2003). Sham animals continued to receive a standard diet.

### Osteoporosis induction

OVX surgery was performed according to previously described protocols (Chen et al., 2018; Khajuria et al., 2012; Lasota & Danowska‐Klonowska, 2004). In the sham group, animals were subjected to the same procedures, but ovaries were only manipulated and not excised. All rats were subjected to surgery with pre‐medication with morphine (3 mg/Kg) and ketamine (10 mg/Kg), and anaesthesia with isoflurane for induction at 5% and O_2_ 100%, and for maintenance at 2–3% and O_2_ 100%. Immediately after the animals recovered from the anaesthesia, they were medicated with meloxicam (1 mg/Kg) intra peritoneal. Subsequently, pain was controlled with tramadol (20 mg/Kg) twice a day for 3–5 days, according to clinical evaluation. Animals' overall health was monitored every day, and they were weighted 3 times a week during the first 2 weeks after the procedure. To confirm the success of the procedures to establish an osteoporotic phenotype, three parameters were adopted, and will be described throughout the article: estrous cycle, uterine horns weight and comparison of bone's volume in rats' tibia between OVX and sham animals.

### Estrous cycle

To confirm the procedure's success, estrous cycle of all animals started to be examined 15 days after the surgery through vaginal cytology. Sixty microlitres of saline solution were injected into rats' vagina with a micropipette, followed by the collection of this fluid. It was placed onto a slide for examination in an optical microscope. The vaginal trophism was classified as estrus, proestrus, metestrus or diestrus. The cell pattern defined whether the animals were under hormonal influence or not (Ceschin et al., 2004). This procedure was repeated every day for 2 weeks, and the analysis was continued for another 7 days in case of incongruous results.

### Apical periodontitis induction

Eight weeks after OVX or sham surgeries, the experimental groups' animals were submitted to apical periodontitis induction. Pre‐medication was administered with morphine (3 mg/Kg) + ketamine (10 mg/Kg), and anaesthesia under isoflurane (for induction at 5% and O_2_ 100%, and for maintenance at 2–3% and O_2_ 100%) was proceeded. Pulp necrosis was induced on animals' mesial roots on left and right mandibular first molars. Dental pulps were exposed by drilling cavities on the mesial portion of the occlusal surface with a 1011 HL round bur in high speed (KG Sorensen) to a depth nearly equal to the bur diameter (1 mm). A #10 endodontic file (Dentsply Maillefer) was then inserted into the root canal until resistance was felt and was used to remove remnants of pulp tissue. Radiographic examination with #10 file in position was performed with Eighteeth Hyperlight Portable DC X‐ray (Changzhou Sifary Medical Technology Co., Ltd.) with 0.10s exposure time, and the images were analysed through Digital xRay System RVG 6200 (Carestream Health) confirming that the mesial root was accessed. The teeth were left open to the oral environment for 3 weeks (Scarparo et al., 2011; Stashenko et al., 1994). The animals were observed daily to evaluate signs of pain or discomfort, and no medication was administered as they all appeared well during the evaluation period.

### Root canal treatment

Twenty‐one days after inducing apical periodontitis, endodontic treatment was conducted on the mesial canals of the left and right lower first molars of experimental groups' animals, following a standard protocol. The animals were anaesthetised as described above.

The working length was determined by an electronic apex locator (EAL) (Dpex III, WoodPecker, Guilin, Guangxi, China) with a #10 endodontic manual K file (Dentsply/Maillefer) 0.5 mm short of the apical foramen and confirmed by radiographic examination. Root canal preparation was performed through serial technique with size 20.04, 25.04 and 30.04 NiTi rotary files (Jara et al., 2018) (Sequence Baby File, MK Life) powered by an electric motor (Reciproc Gold, VDW) driven at a speed 400 rpm and a torque 2 N/cm, as recommended by the manufacturer. Irrigation was conducted with 2 mL of 2.5% NaOCl at the beginning of the procedure and after each instrument change, along with suction. At the end of preparation, irrigation was performed with 2 mL of 17% trisodium EDTA (Biodinâmica, Ibiporâ, PR, Brazil), activated for 3 minutes with a #15 manual K file (Dentsply/Maillefer, Ballaigues, Switzerland). Final irrigation was performed with 2 mL of saline.

Root canals were dried with absorbent paper points. Subsequently, a gutta‐percha master point 30.04 (Dentsply Indústria e Comércio Ltda.) was inserted inside the root canal, respecting its respective working length, and a radiographic examination was proceeded to confirm its adequacy. The root canals were filled through the single‐cone technique, using the gutta‐percha master point and an epoxy resin‐based sealer (Sealer Plus, MK Life). The filling material was sectioned to the cervical level with a heated clinical probe (SSWhite Duflex) and vertically condensed. Cavities were prepared with acid etching (Gluma Etch 35% Gel, Kulzer) followed by an adhesive system (Ambar, FGM) and restored with composite resin (Filtek Z250, 3 M), according to the manufacturer's instructions.

All procedures were performed by the same operator, an experienced and previously trained endodontist, without information about the group to which the animal belonged. Animals were weighed every 2 days for the first week after the procedure, and then once a week. They were also clinically observed for signs of well‐being.

### PBM

Immediately after root canal treatment, the groups OVX/LLL and OVX/GEM received PBM protocols according to Table 1. These protocols were based on previous studies (Mostafavinia et al., 2017; Ribeiro et al., 2022; Romão et al., 2015; Thieme et al., 2020). Groups OVX and sham received simulated application, but with a switched‐off device. All applications were performed 3 times a week until the day of euthanasia (14 or 28 days after endodontic treatment), following the biosafety rules, and under anaesthesia with isoflurane according to previously described protocols. In this manner, groups OVX/LLL 14 and OVX/GEM14 received 6 PBM applications, while OVX/LLL28 and OVX/GEM28 groups received 12 PBM applications.

**TABLE 1:** Photobiomodulation parameters for each group.

Protocol	LLL	GEM
	IO 100 J/cm^2^	EO 6.11 J/cm^2^
Wavelengh (nm)	808 nm ± 10 nm	810 nm + 980 nm (50%/50%)
Operating mode	Continuous	Pulsed
Frequency (Hz)	~50/60 Hz	50 Hz
Pulse duration (ms)	Continuous	2 ms
Duty cicle (%)	–	10%
Polarization	Yes	No
Power (W)	0.1 W	1 W
Average power (mW)	100 mW	1000 mW
Irradiance (W/cm^2^)	3.33 W/cm^2^	2.03 W/cm^2^
Total radiance energy (J)	3 J	30 J
Spot size (cm^2^)	0.03 cm^2^	4.91 cm^2^
Beam shape	Round	Round
Beam profile	–	Gaussian
Area irradiated (cm^2^)	0.03 cm^2^	4.91 cm^2^
Exposure duration (s)	30 s	30 s
Location	Intraoral/periapical area	Extraoral
Number of points irradiated	1	1
Application technique	Contact	Contact
Frequency of treatment sessions	3×/week	3×/week

Group OVX/LLL photobiomodulation therapy was delivered with a continuous gallium‐aluminium‐arsenium (GaAlAs) diode laser (Laser DMC Therapy EC, DMC ABC Equipamentos). Irradiation was performed perpendicularly to the apical regions of the endodontically treated teeth (right and left lower first molars).

Pulse diode laser (Gemini®, Azena Medical, LLC, distributed by Ultradent Products, Inc.) with dual wavelength 810 + 980 nm was used for PBM on the OVX/GEM group. It was performed in contact with the skin of the right and left cheeks, at a point near the mandibula's lower base.

### Euthanasia

**FIGURE 1:**
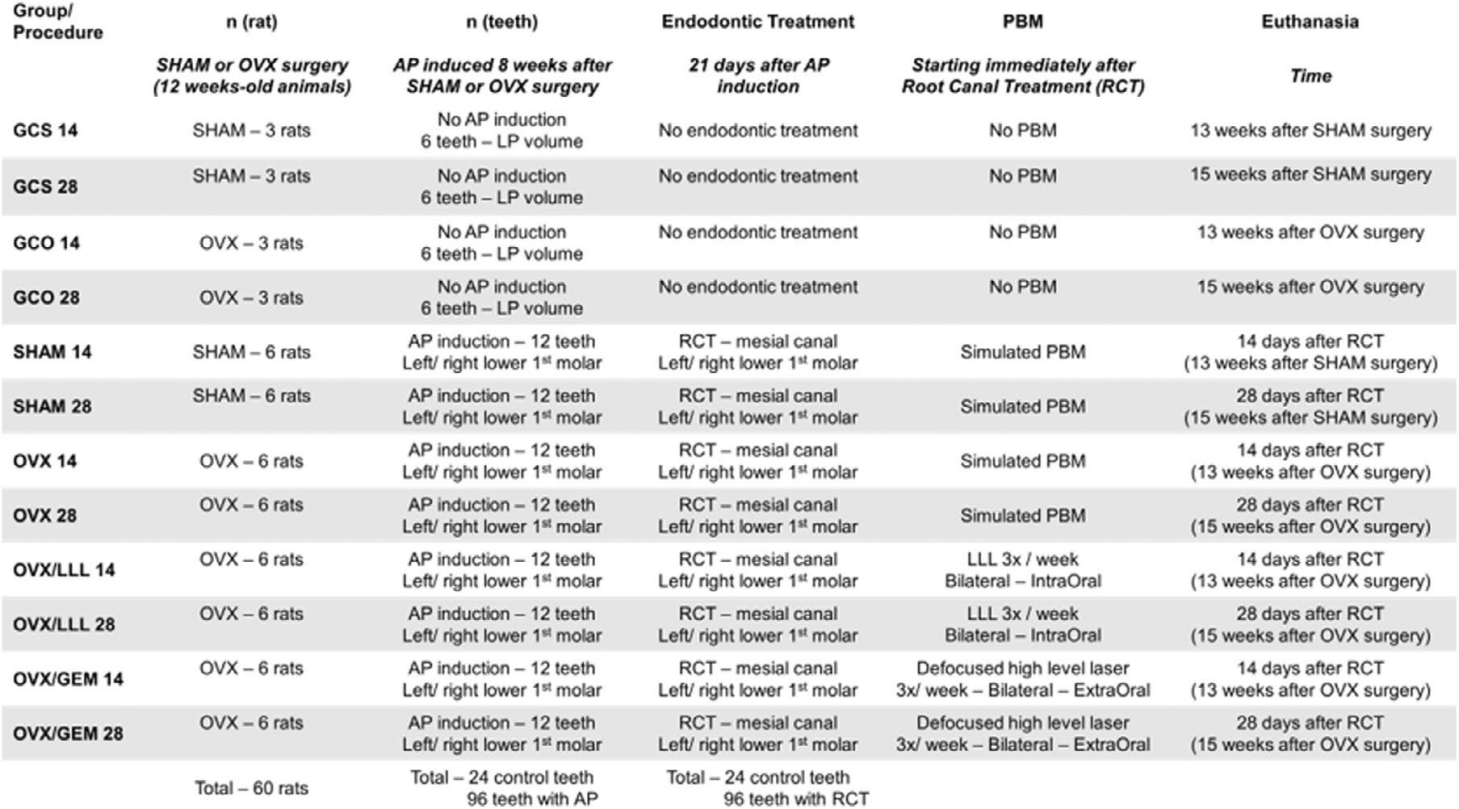
Sequence of procedures and times adopted for control and experimental groups.

**FIGURE 2:**
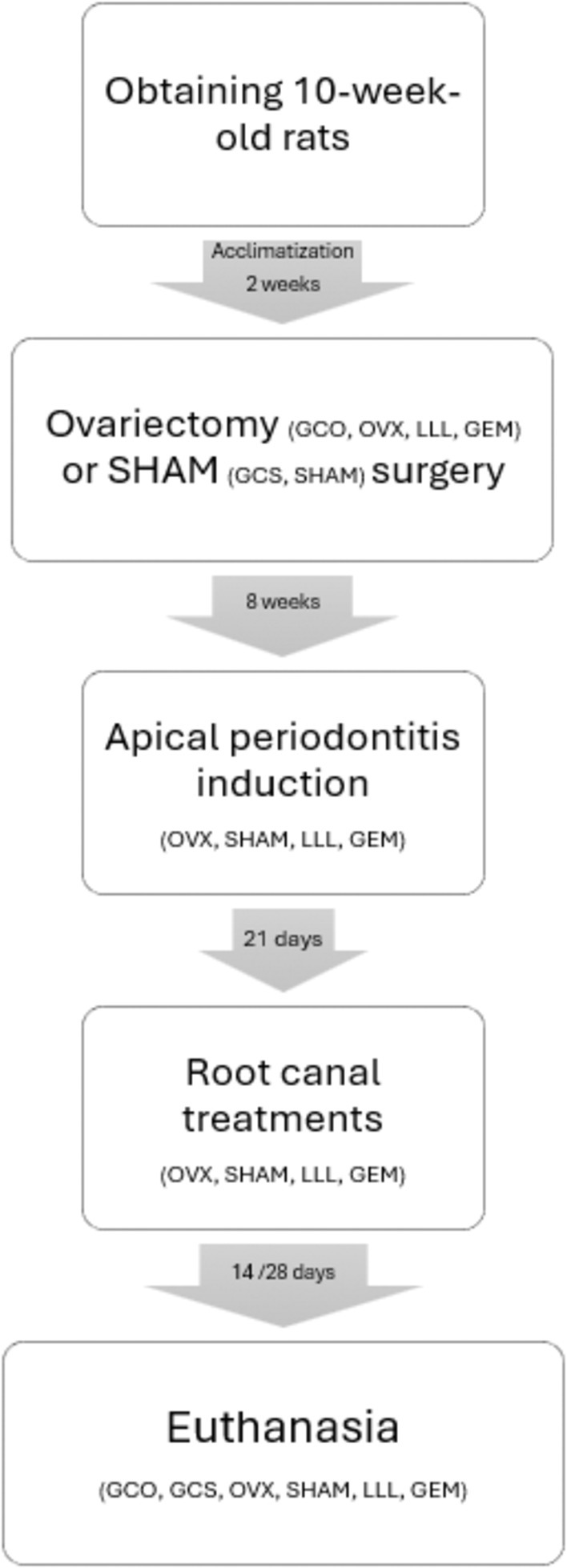
Flowchart showing the sequence across time points for each group, from obtaining the animals to their euthanasia.

After endodontic treatments, half of the sample was euthanized within 14 days. The other half continued receiving PBM or sham application until a 28‐day period, when they were euthanized. Euthanasia was performed using an overdose of inhalational anaesthetic (isoflurane). Control groups were euthanized, respecting the time of the experimental groups (13 and 15 weeks after OVX or sham surgeries). Hemi‐mandibulae and tibias were collected and placed in a bottle containing 20 mL of formaldehyde, duly identified. The presence of intact teeth coronary sealing was assessed at the time of animal euthanasia. Furthermore, uterine horns were removed and weighed on a precision scale to verify the difference from OVX (atrophied) to sham (regular) rats. Figure 1 summarizes the sequence of procedures and the respective times adopted for control and experimental groups. Figure 2 shows the sequence across time points for each group, from obtaining the animals to their euthanasia.

### X‐ray computed microtomography analysis (μCT)

Samples of mandibula and tibia were washed with distilled water and placed in a rotary stage to be evaluated via μCT (MicroCT.SMX‐90 CT; Shimadzu Corp.). Images were obtained in a 360° rotation operated at 70 kV, 100 mA. They were then reconstructed in the InspeXio SMX‐90CT software program (Shimadzu Corp.) with a 17 μm voxel size in images with a 1024 × 1024 pixel resolution and a 10 μm thickness, resulting in 536 images per sample. Images were analysed in an image software program [CTAn software (V1.15.4.0; Bruker)].

Each hemi‐mandibula image was used for the measurement of periodontal ligament (control groups) or volume of periapical bone resorption (experimental groups) around the mesial root of the first molars. Delimitation of the periodontal ligament or the AP lesions was performed in the sagittal plane by a customized delimitation of the region of interest (ROI), adjusted manually in 3 by 3 sections, to ensure the inclusion of the entire bone loss volume at the mesial root of the rat's first molar. Each ROI started with the first sagittal slice of the periapical ligament or the AP lesion and continued to the distal region, ending when it reached the first central root. A colour threshold was manually adjusted and set in at 0–160/190 to all mandibula. Furthermore, endodontically treated canals received a score based on the apical limit of endodontic treatment: (1) adequate apical limit; (2) overflow of endodontic sealer beyond the foramen; (3) overflow of guttapercha and endodontic sealer beyond the apical foramen or filling above the appropriate apical limit.

Tibia images were analysed to demonstrate their osteoporotic or healthy phenotypes. When proceeding with the tibia's analysis, ROI was established as a cubic area of 2 × 2 × 2 mm, starting 1 mm below and 1 mm distal to the epiphyseal plate, inside the trabecular bone. Colour threshold was set on a self‐adjusting scale at 120/130‐255. With the purpose of demonstrating the success of the procedures to induce an osteoporotic phenotype, the following parameters were measured: bone volume fraction (BV/TV), trabecular thickness (Tb.Th), trabecular separation (Tb.Sp), and trabecular number (Tb.N) (Bonnet et al., 2009; Bouxsein et al., 2010).

All the measurements were performed by a trained examiner, blinded to the group the sample belonged to, and submitted to an interclass correlation coefficient (ICC) test prior to the analysis.

### Statistical analysis

All analyses were performed in the SPSS® statistical package (IBM Corp. IBM SPSS Statistics for Windows, Version 25.0.). Data from body weight, uterine weight, tibia bone fraction, trabecular thickness, separation and number, and volume of AP or LP were presented as means ± standard deviation of the means. In order to control for type I error inflation when assessing AP volumes, we fitted a generalized estimating equations (GEE) model with two factors (group and time) plus an interaction term. All pairwise analyses were adjusted for multiple comparisons with the Benjamini–Hochberg (BH) test. Chi‐square test was used to compare the apical limit scores of endodontic treatments between groups. anova was used to evaluate the impact of each of the variables tested on the volumes of apical periodontitis. A *p* value less than 0.05 was considered statistically significant. Graphical illustration was generated with GraphPad Prism, v.5.0 (GraphPad Software, Inc.).

## RESULTS

Five animals were lost during experimental procedures, due to cardiorespiratory arrest: one in group GCO14, two in SHAM14, one in SHAM28 and one in LLL28. There was a coronary fracture of the right molar of one of the animals in the OVX28 group, so this sample had its analysis interrupted in the middle of the experiment. At the time of euthanasia, 100% of the coronary seals were intact.

**FIGURE 3:**
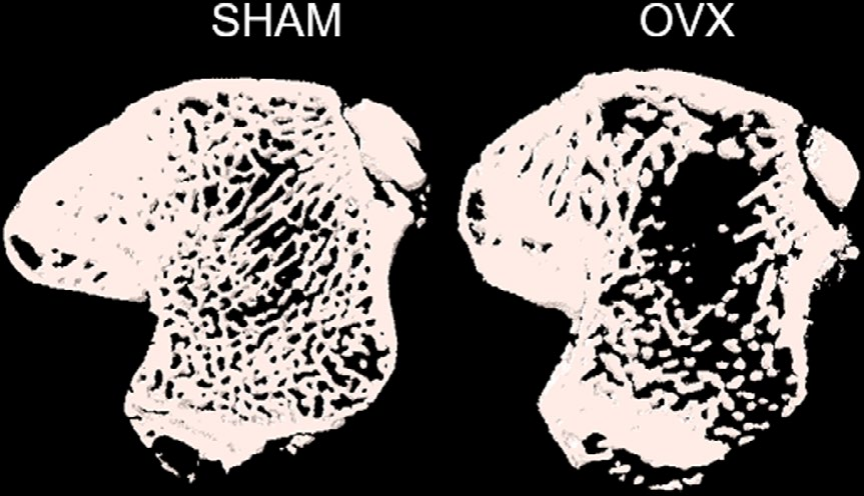
Representative images of volumetric analysis of post‐treatment tibias, obtained by μCT imaging. Reconstructed with CTVol software (V1.15.4.0; Bruker).

The success of OVX was confirmed by monitoring the oestrous cycle, body weight, weight of uterine horns at euthanasia and data regarding the bone density of animals' tibias. Regarding the estrous cycle, at the end of 4 weeks from the OVX or sham surgery 100% of the OVX operated animals were not cycling any longer as they stabilized in diestrous during the analysis. From the sham‐operated animals, all of them presented normal estrous cycle during the analysis, oscillating phase between proestrus, estrus and metestrus. The mean body weight at the beginning of the experiments of the sham and OVX animals was respectively 250.56 g ± 17.17 and 251.00 g ± 17.16 (*p* = 0.928). At euthanasia, the mean body weight for sham and OVX animals was 290.60 g ± 20.19 and 357.05 g ± 25.77, respectively (*p* < 0.0001). Uterine horns' weights from sham‐operated animals were significantly higher than those of the OVX groups (*p* < 0.0001) (Table 2). The fraction bone volume (BV/TV%) and trabecular number (Tb_N) in the medullary component of tibias were significantly lower (*p* < 0.0001), while trabecular separation (Tb_Sp) and thickness (Tb_Th) were significantly higher (*p* < 0.0001 and *p* = 0.020, respectively) in the OVX animals, compared to the sham‐operated ones (Table 2). Figure 3 illustrates the results obtained for sham and OVX tibias.

**TABLE 2:** Uterine horns' weight (g) and bone parameters (%, 1/mm and mm) observed for tibias' analysis.

Group (*n* = rats)	Uterine horn	Tibial analysis			
	Weight (g)	BV/TV (%)	Tb_N (1/mm)	Tb_Sp (mm)	Tb_Th (mm)
	Mean ± SD	Mean ± SD	Mean ± SD	Mean ± SD	Mean ± SD
SHAM (15)	0.7192 ± 0.364	37.27 ± 3.10	0.24 ± 0.02	2.59 ± 0.20	1.52 ± 0.09
OVX (40)	0.1355^a^ ± 0.036	14.22^a^ ± 4.40	0.08^a^ ± 0.03	7.57^a^ ± 2.26	1.60^b^ ± 0.10

Abbreviations: BV/TV, bone fraction volume; Tb_N, trabecular number; Tb_Th, trabecular thickness; Tb_Sp = trabecular separation.

^a^ Different from SHAM (Students' *t* test; *p* < .0001).

^b^ Different from SHAM (Students' *t* test; *p* < .05).

The general average of the intraclass correlation coefficient (ICC) obtained when analysing 25% of the mandible and tibia μCT images twice was 0.9695, which is considered excellent replicability (Fleiss, 1986). Regarding the score of endodontic treatment apical limit, there was no significant difference in quality scores between the groups (*p* = .058).

**FIGURE 4:**
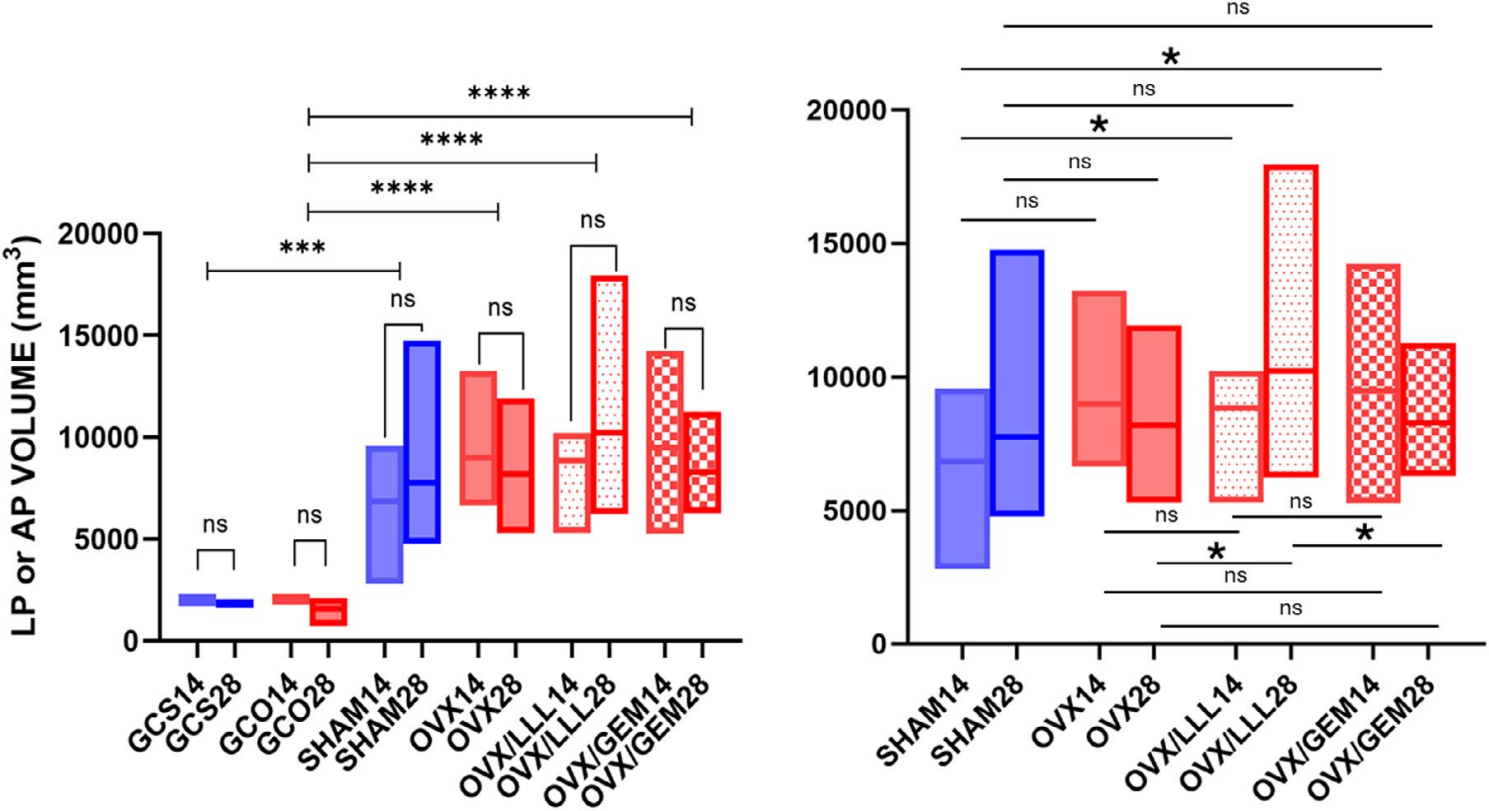
LP or AP volumes (mm^3^) observed for each group using μCT analysis. *p < .05. ***p < .001. ****p < .0001. Graphic illustration generated with GraphPadPrism, v.5.0. (GraphPad Software, Inc.).

**FIGURE 5:**
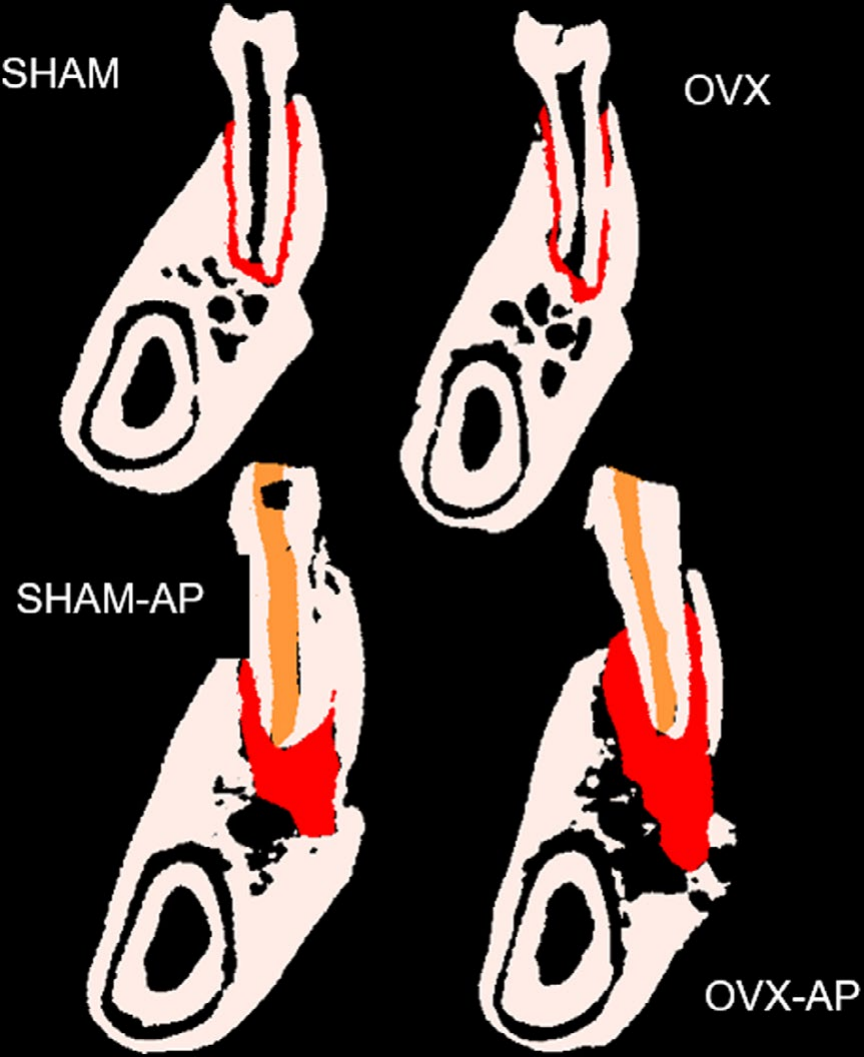
Representative images of volumetric analysis of post‐treatment periodontal ligament or AP, in mm^3^, obtained by μCT imaging. Reconstructed with CTVol software (V1.15.4.0; Bruker).

Data regarding the volume of periodontal ligaments and apical periodontitis are shown in Table 3 and Figure 4. Control groups showed periodontal ligament volumes statistically lower than apical periodontitis' volumes in experimental groups (*p* < .0001). Within each group, there was no statistically significant difference in LP and AP volumes between the two evaluation times (13 and 15 weeks after OVX or sham surgery for control groups, and 14 and 28 days after endodontic treatment for experimental groups) (*p* > .05). There was no significant difference between the experimental groups in relation to APs volume (*p* > .05), except for the lower values observed in SHAM14 when compared to OVX/LLL14 (*p* = .030) and OVX/GEM14 (*p* = .034) groups, and higher values obtained for OVX/LLL28 when compared to OVX28 (*p* = .018) and to OVX/GEM28 (*p* = .022). Grouping all ovariectomized experimental animals (ALL OVX) and comparing them with the sham animals, there was a statistically significant difference in relation to the volume of AP, with greater values for the ALL OVX group (*p* = .009). Figure 5 illustrates the obtained results for hemi‐mandibulae. When the effect of the variables on the outcome was jointly evaluated through anova, ovariectomy was the only one that showed a significant influence on the increase in AP volume (*p* = .004). Time (14 or 28 days), apical limit score for endodontic treatments (1, 2 or 3) and use or source of PBM (no PBM, LLL or GEM) did not demonstrate significant influence on the outcome (Table 4).

**TABLE 3:** LP or AP volumes (mm^3^), expressed in means and standard deviation, observed for each group using μCT analysis. Comparisons between groups according to generalized estimating equations (GEE) and adjusted for multiple comparisons with Benjamini‐Hochberg (BH).

GROUP/TIME	GCS	GCO	SHAM	OVX	OVX/LLL	OVX/GEM
14 days	2.079 ± 0.254 (*n* = 6) a, A	2.012 ± 0.030 (*n* = 4) a, A	6.849 ± 2.070 (*n* = 8) a, B	8.971 ± 2.131 (*n* = 12) a, B	8.838 ± 1.287 (*n* = 12) a, B, *	9.470 ± 2.685 (*n* = 12) a, B, *
28 days	1.855 ± 0.164 (*n* = 6) a, A	1.573 ± 0.629 (*n* = 6) a, A	7.741 ± 3.105 (*n* = 10) a, B	8.173 ± 1.784 (*n* = 11) a, B	10.223 ± 3.208 (*n* = 10) a, B, #	8.285 ± 1.521 (*n* = 12) a, B, &

*Note*: Lower case letters: comparison between times within the same group (*p* < .05). Capital letters: comparison between control and experimental groups (*p* < .05). *: different from SHAM group (*p* < .05). #: different from OVX group (*p* < .05). &: different from OVX/LLL group (*p* < .05).

Abbreviations: AP, apical periodontitis; LP, periodontal ligament; SD, standard deviation.

**TABLE 4:** Analysis of variables on their impact on AP volume outcome (anova).

Variables		Group SHAM (*n* = 18)	Group ALL OVX (*n* = 69)	Mean ± std. error	*p* Value (anova)
Osteoporosis	No	18	0	7.827 ± 657.20	.004
	Yes	0	69	9.090 ± 315.55	
Esc_Qual	1	5	18	7.839 ± 490.76	.064
	2	7	10	9.214 ± 570.83	
	3	6	41	8.812 ± 343.30	
Time (days)	14	8	36	8.685 ± 335.64	.509
	28	10	33	8.581 ± 389.29	
PMB	No	0	23	8.466 ± 489.37	.201
	LLL	0	22	9.657 ± 504.99	
	GEM	0	24	9.070 ± 470.89	

*Note*: Esq_Qual: apical limit score for the endodontic treatment (1 = satisfactory; 2 = sealer extra; 3 = sealer + gutpercha extra).

**FIGURE 6:**
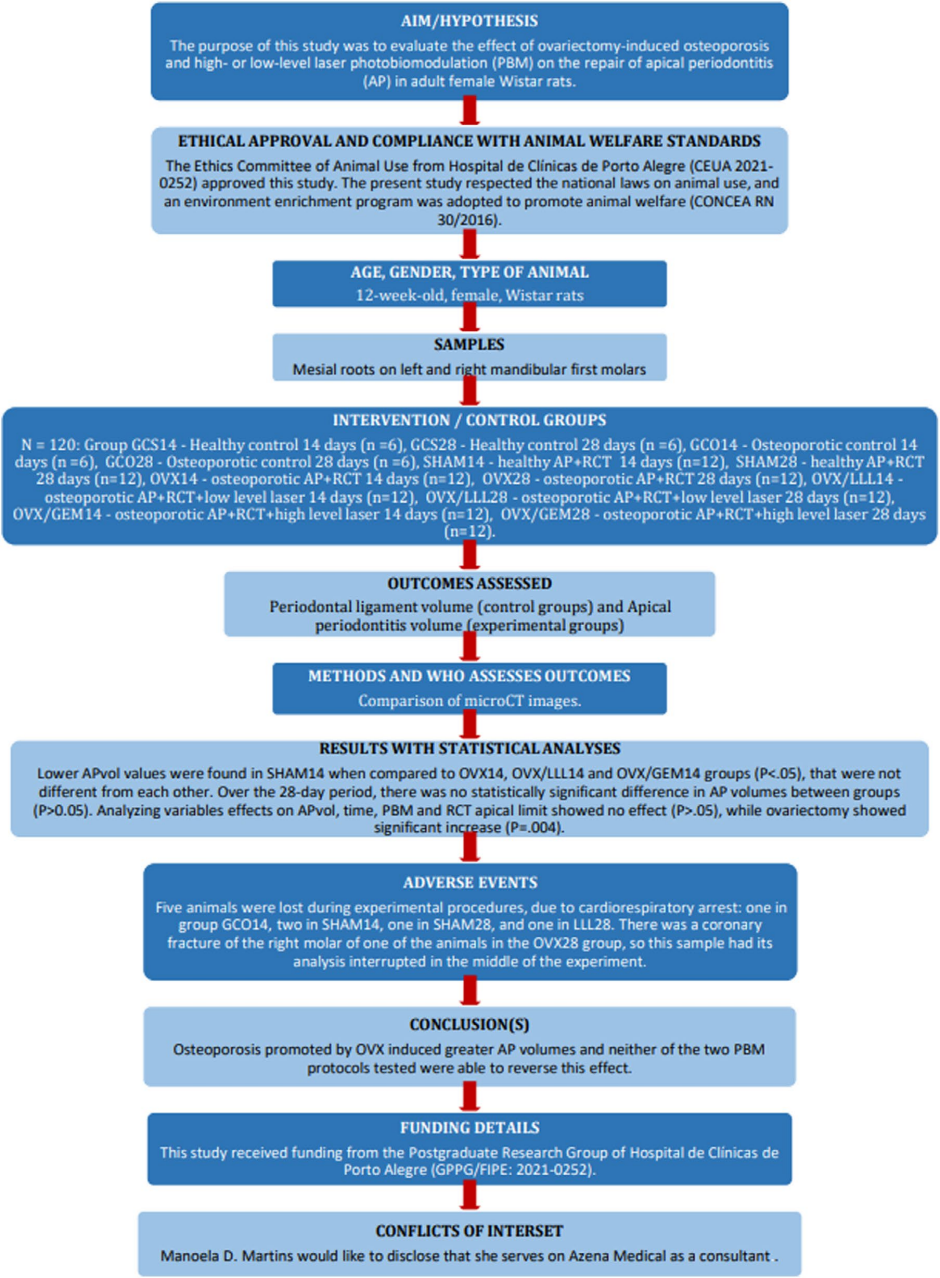
Flowchart of the study according to PRIASE 2021 guidelines.

A flowchart containing the most relevant information from this study in accordance with Preferred Reporting Items for Animal studies in Endodontology (PRIASE) 2021 guidelines (Nagendrababu et al., 2021) is shown in Figure 6.

## DISCUSSION

This is the first study that evaluated the effect of photobiomodulation on apical periodontitis volume after endodontic treatment of necrotic teeth in ovariectomized rats. Considering that both osteoporosis (Wright et al., 2014) and apical periodontitis (Tibúrcio‐Machado et al., 2021) are diseases with high prevalence in the world, and that both involve similar and synergistic processes (Lerner, 2006; Liu et al., 2010), it is extremely important to search for therapeutic approaches that can help restore oral (and consequently systemic) health of individuals. The literature is full of pre‐clinical studies that prove that apical periodontitis is larger in osteoporotic animals (Brasil et al., 2017; Gilles et al., 1997; Gomes‐Filho et al., 2015; Guan et al., 2020; Liu et al., 2010; Qian et al., 2020; Romualdo et al., 2018; Wayama et al., 2015; Xiong et al., 2007; Zhang et al., 2011), and that their effects are quite relevant, with an increase in systemic inflammatory cytokines (Qian et al., 2020; Romualdo et al., 2018; Zhang et al., 2011). Since PBM therapy is widely accepted and has been studied for promoting and accelerating oral tissues healing (Berni et al., 2023; Dompe et al., 2020; Son et al., 2017), the hypothesis of the present study was that it would be capable of accelerating AP's healing process after endodontic treatment, which would be confirmed if their volumes were smaller in the irradiated groups. Both 14 and 28 days after endodontic treatment, AP in ovariectomized rats showed a tendency to have larger volumes, regardless of the use of PBM. The results confirmed the impact of osteoporosis on AP; however, PBM was not effective in reducing this impact, even causing greater volumes of apical periodontitis for the group irradiated with low‐power laser for a longer period (28 days).

Two clinical studies were previously conducted to assess the effect of PBM on periapical healing in healthy patients and found that adjunct therapy with low‐level laser accelerated the healing process of AP 9 months after endodontic treatment (Das et al., 2023; Shah et al., 2021). In our study, PBM groups did not achieve better results compared to OVX in relation to the parameter analysed. There is a major reason that might explain the differences between the present and the previous studies: the latter was conducted in healthy patients. On the other hand, in the osteoporosis model adopted in the present study, the bone process of reabsorption/neoformation is significantly altered and harmed, since there's an imbalance in the RANK/RANKL/OPG system that increases the proliferation of osteoclasts and, consequently, stimulates bone resorption (Khosla & Riggs, 2005). Furthermore, a review of PBM parameters conducted by Zein et al. (2018) postulated that tissues with few mitochondria (like bone) tend to show PBM effective results when a higher fluence is applied. It could be hypothesized that the ineffective results obtained in the present study are related to PBM parameters, which are broad, complex and heterogeneous. Nonetheless, the applied parameters in OVX/LLL groups are not new and have been shown to be effective in previous studies, with enhanced bone healing and mineralization on alveolar region both in animal (Ribeiro et al., 2022) and human (Romão et al., 2015) experimental models. Since these studies were conducted on healthy individuals, it is very likely that the unfavourable results found for the use of LLL in the present study originated from the correlation with osteoporosis. Mostafavinia et al. (2017) tested the effect of LLL on tibial defects in osteoporotic rats and failed to find positive responses and attributed this outcome to the hypothesis of a profound systemic effect of osteoporosis, affirming that osteoporotic bone tissue could not respond properly to laser biostimulation. Likewise, our study didn't find a statistically significant difference in AP volumes between OVX and OVX/GEM groups, in which PBM was performed using a defocused high‐power laser. Since it works with two different wavelengths, thus increasing the chance of reaching deeper tissues such as bone, we hypothesized that it could render positive results. It could be inferred that the lack of effect is due to the low fluence adopted, since favourable results with this dose were obtained in a study that aimed to improve oral mucositis (Thieme et al., 2020); so possibly a higher fluence would be necessary to achieve bone repair.

The present study confirmed the effectiveness of AP induction, since periodontal ligament volumes in both sham and OVX control groups were significantly smaller in relation to the bone loss adjacent to the roots whose canals were exposed to the oral environment. This methodology has already been widely used, and is in agreement with a previous report (Frazão et al., 2023). Concerning the difference between AP volume in sham and OVX experimental groups, a tendency towards greater AP volumes was observed in the ovariectomized group, although this difference was not statistically significant in the paired analysis between groups. These results are in agreement with several previous studies (Gomes‐Filho et al., 2015; Guan et al., 2020; Liu et al., 2010; Qian et al., 2016, 2020; Romualdo et al., 2018; Xiong et al., 2007; Zhang et al., 2011) which, although did not perform endodontic treatment, proved that ovariectomized animals exhibit larger apical periodontitis lesions. A possible explanation for the lack of statistical difference is the high standard deviation observed for the groups. With a high standard deviation, a larger sample might have been necessary for the apparent tendency towards difference (which can be seen by observing mean and medians values in groups, in Table 3) to be statistically proven. Our calculation was based on a study that evaluated healthy animals (Çırak et al., 2018), and perhaps, in the presence of a systemic condition such as osteoporosis, the imbalance of bone metabolism may be responsible for the variability in results.

An interesting finding was that a tendency for higher AP volumes in the ovariectomized groups were found in the analysis performed 14 days after endodontic treatment. There is a possible explanation for this result. The hypothesis has to do with the imbalance in RANK/ RANKL/ OPG system in osteoporotic animals. Some studies evaluated the number of positive cells for RANKL and for TRAP enzyme, which is a biomarker for osteoclasts. Their results showed that this number (and the consequent bone resorption) peaks 14 days after PA induction, and that it tends to decrease as the weeks go by (Guan et al., 2020; Qian et al., 2016; Zhang et al., 2007). This trend was different for the low‐level laser irradiation group (OVX/LLL). AP volumes were significantly higher in the 28‐day period compared to both the OVX and OVX/GEM groups. It therefore appears that the LLL influenced increasing the volume of AP. A possible reason for this negative effect is the so‐called Arndt‐Schultz law, which states that ‘for every substance, small doses stimulate, moderate doses inhibit, and large doses kill’ (Huang et al., 2009). As the number of photons delivered by the laser increases beyond a particular level, the cellular stimulation disappears, and inhibition and cellular damage occurs; ATP reserves within the cell begin to be depleted by excessive doses of light, compromising the positive cellular function (Zein et al., 2018). Thus, maybe the 100 J/cm^2^ fluence adopted in this group was excessive.

Apart from paired and adjusted comparison between groups and due to the lack of PBM significant effect on AP volume's reduction, the average data from samples from all the OVX animals was obtained and compared with the results from the sham group. The analysis showed no impact of time post‐endodontic treatment and PBM on AP volume. The only variable that proved to be significant in increasing PA volume was ovariectomy, confirming results of previous research (Brasil et al., 2017; Gomes‐Filho et al., 2015; Guan et al., 2020; Qian et al., 2016, 2020; Romualdo et al., 2018; Wayama et al., 2015; Xiong et al., 2007).

The chosen preclinical experimental model was the ovariectomized female rat. It is a well‐established method, approved by the Food and Drug Administration, that causes a decrease in oestrogen production, simulating the condition of postmenopausal ovaries, and is commonly used in the health field to study the effects of osteoporosis (Kalu, 1991). It is known that OVX alters animals' estrous cycle, preventing ovulation, promotes an increase in body weight and uterine horn's atrophy. Therefore, monitoring these parameters was essential to confirm the success of the surgical procedure. Our results are in accordance with previous studies, which showed that ovariectomized rats remain in diestrous during estrous cycle analysis (Brasil et al., 2017; Gomes‐Filho et al., 2015; Wayama et al., 2015; Xiong et al., 2007) and present greater body weight and horns' uterine atrophy (Brasil et al., 2017; Gomes‐Filho et al., 2015; Romualdo et al., 2018; Silva et al., 2020). Furthermore, in the present study, images from tibias were obtained in microCT and showed that OVX was able to reduce bone fraction volume and trabeculae number, and raise trabeculae thickness and separation 13 or 15 weeks after the surgical procedure, indicating that the expected effect was obtained. These results are in agreement with previous studies (Romualdo et al., 2018; Silva et al., 2020; Zhang et al., 2007). On the other hand, there is a lot of variability in methodologies in relation to the time needed after ovariectomy for the osteoporotic bone phenotype to be achieved, especially in relation to the maxillary bones, which are subjected to mechanical load during mastication that influences its bone mass and architecture (Mavropoulos et al., 2007). A meta‐analysis conducted on this topic revealed radiologic microarchitectural change consistent with osteoporosis in the mandible of OVX rats, but stated that, although the post‐OVX period required for bone structural changes to occur in the mandible is not conclusively known, longer post‐OVX periods show more notable decrease in bone mineral density (Lee et al., 2019). They also suggest the use of 12‐week‐old rat models to proceed with OVX, since they are, at this age, sexually mature, eliminating the possibility of bone loss and disease caused by aging, and respond much more rapidly to OVX than aged rats, reducing the time and cost of the study. A previous systematic review that aimed to address whether osteoporosis induction would impair alveolar bone repair in animal models suggested, for further studies, nine recommendations regarding animal characteristics, diet, experimental periods and methods to assess the success of OVX, in order to standardize and minimize the variability among studies (Só et al., 2021). These methodological recommendations were rigorously followed in the present experimental study, and the osteoporotic animal model was evidenced by the results of estrous cycle, weight gain, tibia bone volume and the difference between SHAM and all OVX AP volume. Another point that should be highlighted is the role of possible confounders that are present when conducting studies in humans. Both osteoporosis and periodontitis are quite prevalent diseases in the aging population and have some common risk factors like smoking, poor nutritional status, age, immune deficiency, gender, socioeconomic status, genetics and systemic inflammation (Penoni et al., 2017). These factors contribute to bone loss in both conditions and make it difficult to isolate the specific impact of one disease on the other. By conducting the study on animals, the influence of these confounders is overcome.

The literature is scarce in studies that have performed endodontic treatment of teeth with AP and evaluated their apical repair in rats. Performing endodontic treatment on rat molars is quite challenging, since access is limited, teeth are small, the field of view is restricted and the equipment used needs to be adapted for this purpose (Dammaschke, 2010). Consequently, in the present study, although the apical limits appeared precise in the radiographic analysis, not all endodontic treatments evaluated in μCt were within the appropriate filling limits. Thus, three scores were created to classify the apical limit of endodontic treatments. Analysis of the results revealed that scores did not have an impact on AP volume. This finding is corroborated by a previous clinical study (Ricucci et al., 2016)—they stated that treatment outcome is not affected by extruded sealer as long as the root canals are adequately treated. On the other hand, they differ from the results of the meta‐analysis conducted by Ng et al. (2011), according to whom a long root filling reduced endodontic treatment's odds of success by 62%.

The present study has limitations that may be considered before extrapolation of its results. The first of these concerns is carrying out the study on an animal model. Although the study on rats is consolidated in literature, interpretation of its findings must be careful, since they should not be taken directly to clinical practice. The second is the unique μCT method used to measure outcomes. Histomorphometry analysis would be of great value to confirm the data found and elucidate the degree of inflammatory response of the tissues, as well as immunohistochemical analysis would allow the measurement of markers of bone resorption. Cost limitations postponed these evaluation methods that would be important to assist in the interpretation of our results. The third limitation is due to the anatomy of the teeth used in the present study. They have four roots, mesial and distal are the largest and buccal and lingual are in the furcation region and have a very small length and thickness, which makes endodontic treatment unfeasible (Yoneda et al., 2017). In some samples, however, the induction of AP in the mesial root also ended up inducing bone loss adjacent to the central roots, which may have overestimated the volume of AP measured in the images. Finally, the fourth and perhaps most relevant limitation is the impossibility of measuring AP before carrying out endodontic treatments and PBM therapy, which made intra‐group comparison unfeasible and only allowed comparison between groups. The control groups allowed us to confirm the AP induction method but were unable to elucidate whether the volume of AP in untreated canals would be greater. Future studies with an increase in the sample number would make it possible to create control groups where AP was induced and the root canals remained untreated, to compare their results with the treated groups. Another perspective for future studies is to perform in vivo μCT analysis. It would provide the high resolution of μCT while allowing for longitudinal studies of bone morphology (Bouxsein et al., 2010). It would be, thus, an ideal strategy for evaluating periapical tissues repair. Furthermore, different parameters of photobiomodulation therapy need to be tested for their effect on the repair of AP lesions, as the literature is quite controversial in this regard.

## CONCLUSIONS

This study evaluated the effects of osteoporosis and two PBM protocols in the repair of AP for the first time in the literature. Some conclusions can be drawn within the limitations of this preclinical study. Time after endodontic treatment did not significantly impact AP volumes, as well as the apical limit of endodontic treatments. Ovariectomy‐induced osteoporosis in Wistar rats tended to induce greater AP volumes and neither of the two PBM protocols tested was able to counteract this effect.

## AUTHOR CONTRIBUTIONS

Carolina Horn Troian‐Michel: Conceptualization, Methodology, Formal analysis, Investigation, Data Curation, Writing—Original Draft, Visualization, Project administration; Bruna Barcelos Só: Conceptualization, Methodology, Investigation; Lauren Frenzel Schuch: Methodology, Investigation; Grazielle Oliveira Stelter: Methodology, Investigation; Tuany Rafaeli Schmidt: Methodology, Investigation; Joana Letícia Schorr: Methodology, Investigation; Daniela Campagnol: Methodology, Investigation; Tuane Nerissa Alves Garcez: Conceptualization, Resources; Gabriel Barcelos Só: Formal analysis; Thalita Ayres Arrué: Methodology, Investigation; Theodoro Weissheimer: Methodology, Investigation; Manoela Domingues Martins: Conceptualization, Resources, Supervision; Marcus Vinicius Reis Só: Data curation, Writing—Review and Editing, Supervision.

## FUNDING INFORMATION

This study received funding from the Postgraduate Research Group of Hospital de Clínicas de Porto Alegre (GPPG/FIPE: 2021‐0252). The authors express their gratitude to the Coordination of Improvement of Higher Education Personnel (CAPES, Finance Code 001) in Brazil. C.H.T.M., L.F.S. and T.R.S. are recipients of fellowships.

## CONFLICT OF INTEREST STATEMENT

Manoela D. Martins would like to disclose that she serves at Azena Medical as a consultant.

## ETHICS STATEMENT

The Ethics Committee of Animal Use from Hospital de Clínicas de Porto Alegre (CEUA 2021‐0252) approved this study.

## Data Availability

The data that support the findings of this study are available from the corresponding author upon reasonable request.
